# Beyond the Brushstrokes—Illuminating Patterns and Interactions to Find Order in Complex Systems

**DOI:** 10.3201/eid3113.AC3113

**Published:** 2025-05

**Authors:** Duncan MacCannell, Bronwyn MacInnis, Scott Santibanez

**Affiliations:** Centers for Disease Control and Prevention, Atlanta, Georgia, USA (D. MacCannell, S. Santibanez); The Broad Institute of MIT and Harvard, Cambridge, Massachusetts, USA (B. MacInnis)

**Keywords:** Advanced Molecular Detection, genomics, epidemiology, Vincent van Gogh, The Starry Night

**Figure:**
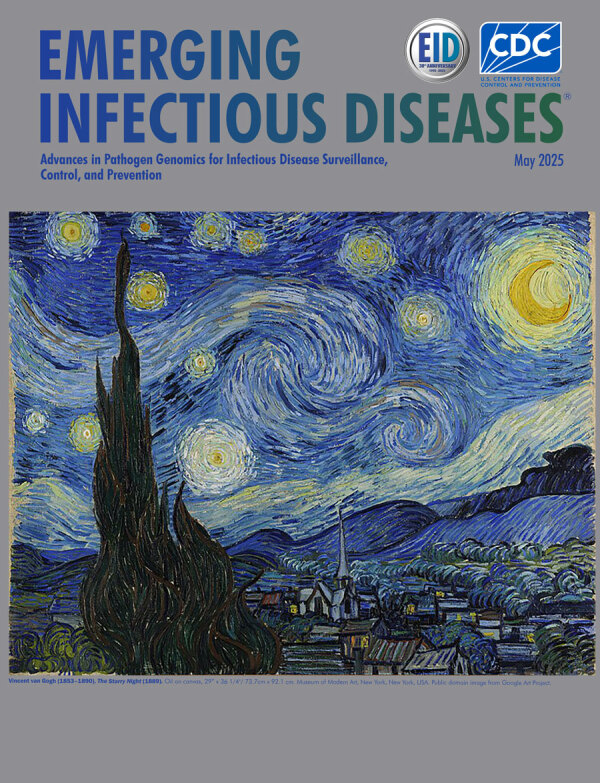
On the cover: **Vincent van Gogh (1853–1890), *The Starry Night* (1889).** Oil on canvas, 29 in × 36¼ in/73.7 cm × 92.1 cm. Museum of Modern Art, New York, New York, USA. Public domain image from Google Art Project.

Vincent van Gogh’s *The Starry Night* is widely considered a postimpressionist masterpiece and is one of the most recognizable pieces of art in modern history. It also presents a metaphor for public health innovation, particularly the advances in pathogen genomics and genomic epidemiology that are the central theme of this supplemental issue of Emerging Infectious Diseases.

*The Starry Night* was one of more than 150 paintings that van Gogh produced in 1889 and shared with a doctor friend during a year of recovery and intense personal turmoil in Saint-Rémy-de-Provence. The work captures the swirling clouds and interconnected stars of the night sky with a staccato of short and purposeful brush strokes. Up close, these individual strokes seem chaotic and disjointed, but they resolve into coherent dimensions of time and space when viewed from afar. In pathogen genomics, each genetic sequence, much like a brushstroke, provides only a glimpse of a pathogen’s genomic structure, characteristics, or origins. When viewed through a bioinformatic lens, those sequence fragments can be aligned and assembled into a more complete picture, with further resolution of the genomic landscape, and a new understanding of its features that emerges when each fragment is appreciated in the context of hundreds or even thousands of others.

The painting also confers a sense of movement and unity: the whirling clouds, punctuated by celestial bodies that capture the viewer’s attention and evoke feelings of connection and interaction. Similarly, genomic epidemiology integrates molecular and epidemiologic data to illuminate complex patterns of disease transmission and the interactions among pathogens, hosts, and entire populations. Just as van Gogh used the clouds to connect the stars in his sky, genomic epidemiologists draw links between cases, mapping transmission chains and identifying sources of infection.

The shapes and colors of this painting echo the data visualization methods that have emerged over the past decade to help scientists communicate complex phylogenetic and phylogeographic data to other public health professionals, policymakers, and the public. Visualization tools such as phylogenetic trees, flowcharts, heatmaps, and transmission networks help to translate nuanced genomic data into understandable narratives through color and form.

For van Gogh, this painting also reflects an important period of introspection and discovery, as well as an attempt to capture and interpret his environment. Applied public health and infectious disease research share a similar intention, focused on better understanding and responding to infectious disease threats. In a sense, both represent an effort to find order in complex systems, and to reveal the hidden patterns that connect them.
